# Adaptation and conservation of CL-10/11 in avian lungs: implications for their role in pulmonary innate immune protection

**DOI:** 10.1098/rstb.2023.0425

**Published:** 2025-02-27

**Authors:** Srinivasa Reddy Kunchala, Albert van Dijk, Edwin J. A. Veldhuizen, Henk P. Haagsman, Sandra Orgeig

**Affiliations:** ^1^ Clinical and Health Sciences, University of South Australia, Adelaide SA 5000, Australia; ^2^ Department of Biomolecular Health Sciences, Faculty of Veterinary Medicine, Utrecht University, Utrecht, The Netherlands

**Keywords:** pulmonary innate immunity, avian non-surfactant proteins, avian collectin protein evolution, avian CL-10, avian CL-11, zoonotic respiratory infections

## Abstract

The common avian origin of many zoonotic infections and epidemics warrants investigation into the mechanism of respiratory surface protection in reservoir species such as birds. Our recent molecular investigations on the evolution and pulmonary expression of an ancient family of proteins, the C-type lectins, have revealed unique molecular adaptations in the surfactant proteins avian SP-A1 (aSP-A1), aSP-A2 and aSP-C coupled with the loss of surfactant protein-D (SP-D) in the avian lineage. As surfactant proteins are members of the collectin family, a subgroup of the C-type lectins, an *in silico* search for related non-surfactant collectin proteins (Collectin-10 (CL-10) and Collectin-11 (CL-11)) in the NCBI genome database was conducted to understand their evolution in the avian lineage. In addition, both CL-10 and CL-11 gene expression in the lungs and other organs of zebra finches and turkeys was confirmed by PCR. These PCR-confirmed zebra finch and turkey CL-10 and CL-11 sequences were compared with sequenced and *in silico*-predicted vertebrate homologues to develop a phylogenetic tree. Compared with avian surfactant proteins, CL-10 and CL-11 are highly conserved among vertebrates, suggesting a critical role in development and innate immune protection. The conservation of CL-11 EPN and collagen domain motifs may compensate to some extent for the loss of SP-D in the avian lineage.

This article is part of the theme issue ‘The biology of the avian respiratory system’.

## 1. Introduction

There are major differences between mammals and birds in the anatomy and morphology of the respiratory system, and as a result the airflow is different [1]. The avian respiratory system evolved a unique anatomy and physiology compared with all other air-breathing vertebrates due to flight being part of the avian lifestyle [2]. The unique features include unidirectional airflow entering the air sacs that pump air in one direction through the rigid parabronchi (parabronchial lung), also known as the true lung [3]. In contrast to mammalian lungs, avian lungs cannot undergo compression and expansion, and as a result gas exchange occurs in the narrow, rigid air capillaries. Because of this adaptation, gas exchange in birds occurs with high efficiency to meet the metabolic demands of flight [1,4]. These anatomical and physiological adaptations of the respiratory system are accompanied by adaptations in the pulmonary surfactant system as surfactant composition and function change to suit the respiratory structure and needs of organisms [5,6]. While surfactant in mammals is designed to maintain alveolar stability and prevent end-expiratory collapse in pulsating alveoli, the avian surfactant system is primarily designed to keep the narrow air capillaries open. Hence, avian surfactant does not require the extreme reduction and variation of surface tension that is necessary to facilitate breathing in mammals [7]. For example, chicken and duck surfactants contain a higher percentage of the disaturated phospholipid dipalmitoyl phosphatidylcholine (PC16:0/16:0), but less palmitoylmyristoyl phosphatidylcholine (PC16:0/14:0) and palmitoylpalmitoleoyl (PC16:0/16:1) [5], while the protein composition has also changed significantly (see below). Probably because of these differences, avian lung surfactant possesses a lower capacity to reduce surface tension than mammalian alveolar surfactant [5,8–10]. It has also been proposed that the presence of pulmonary surfactant in the air capillaries of birds may serve a gas exchange function by aiding in the transport of oxygen, as water layers saturated with pulmonary surfactant membranes can transport oxygen much faster than either pure water layers or those consisting of concentrated phospholipid membranes [11]. Recently, it has been proposed that this phenomenon is aided by the trapping of oxygen-rich nanobubbles in the three-dimensional lipo-protein surfactant structures [12]. However, as tubular myelin structures appear to be absent in bird surfactants [5], these theories remain to be tested. In addition, marked immunological differences exist between the avian and mammalian respiratory systems. Birds have a very low number of resident avian respiratory phagocyte (ARP) cells (i.e. macrophages, polymorphonuclear neutrophils and heterophils on their pulmonary surfaces [13,14]) and depend heavily on the infiltration of ARPs in the case of infection [13,15]. This dependency is further supported by the more efficient engulfment and clearance of various air-associated particles by avian macrophages [15]. Regardless of anatomical, morphological and physiological differences between mammals and birds, both need protection of the lung surface from common air-associated antigens.

Like all air-breathing vertebrates, mammals and birds have to contend with similar inhaled respiratory antigens. The antigen requires swift recognition to block the potential infection. This involves both direct binding by innate immune proteins and marking for uptake by phagocytic cells, which are responsible for processing and antigen presentation for clearance by the inflammatory and/or adaptive immune response [16–20]. The collagenous surfactant proteins (surfactant protein-D (SP-D) and surfactant protein A (SP-A)) predominantly protect the human respiratory system [21–24] in association with other innate and adaptive immune components [18,25]. The collagenous C-type lectins consist of four domains—N-terminal region, collagen domain, neck and carbohydrate recognition domain (CRD)—and are classified as the collectin family of proteins [26]. This family includes mannan-binding lectin (MBL) [27], SP-A [28], SP-D [29], Collectin 43 (CL-43) [30], conglutinin [31], Collectin 46 (CL-46) [32], Collectin 10 (CL-10, also known as Collectin liver 1 (CL-L1)) [33], Collectin 11 (CL-11, also known as Collectin kidney 1 (CL-K1)) [34] and Collectin 12 (CL-12, also known as Collectin placenta 1 (CL-P1)) [35,36].

The role of the surfactant collectins in mobilizing these innate and adaptive immune responses requires the collagen domain to interact with myeloid cell surface receptors [37–45]. Specifically, the SP-D collagen domain communicates with the cell surface receptor calreticulin/CD91, leading to an increase in phagocytosis by macrophages as well as stimulation of p38 phosphorylation, NF-κB activation and inflammatory cytokine production [17,46]. Similarly, the SP-A collagen domain interacts with the myeloid cell SP-R210 receptor [47], the Fc receptor, complement (CR1) [48], the C1q receptor [26,49] and also calreticulin/CD91 receptors [50]. These interactions lead to various antigen clearance pathways and the regulation of cellular inflammation, demonstrating the important communication role of the collagen domain in lung protection.

Functional studies have indicated that mammalian SP-D and SP-A have many overlapping immune functions but that SP-D is more active than SP-A against many pathogens [51,52], demonstrating a more efficient aggregation, opsonization and upregulation of cell surface receptors [21,53,54]. This may be explained by a higher affinity of SP-D for carbohydrates present on a wide variety of antigens, resulting in enhanced aggregation and phagocytosis [55–57]. This also suggests that SP-D is a major contributor to pulmonary innate immunity. Additionally, SP-D plays a role in non-respiratory diseases such as cardiovascular disease (CVD) and metabolic homeostasis due to its expression in non-pulmonary tissues [46], supporting a broader function for SP-D. Given the importance of the collectins to pulmonary host defence in mammals and the focus on birds as reservoir species for many zoonotic infections, we have been investigating the molecular evolution and pulmonary expression of these proteins in birds. We have discovered that birds lack SP-D completely and that they possess unique molecular adaptations in the surfactant collectin proteins SP-A1 and A2 [58]. Specifically, they demonstrate a significant reduction in the length of the collagen domain while the N-terminal, neck and CRD domains are highly conserved [58]. Given that birds have lost the SP-D protein completely, lost the collagen domain of SP-A2 and demonstrate a shorter SP-A1 collagen domain compared with mammalian species [36,58], the question arises as to how birds protect their pulmonary surfaces in the absence of important collagenous lectins such as mammalian SP-A and SP-D.

In addition to SP-A and SP-D, some collectin family members, such as MBL, CL-10, CL-11 (only in chicken) and CL-12 were shown to be expressed in the lungs of various vertebrates. Further investigation of the developmental expression of CL-10/11 in humans, mice and zebrafish confirmed a key role for these proteins in embryonic development, innate immune protection in the circulation as well as their expression in human and mouse lungs [34,59–61]. Moreover, *in vitro* mammalian CL-10/11 studies showed their role in innate immune defence [62]. Similarly, investigations of CL-10/11 gene expression confirmed expression in the chicken lungs before and after hatching [63] and in adult bird lungs [36] following pulmonary exposure to infectious bronchitis virus (IBV) and *Escherichia coli* [64], suggesting their contribution to chicken pulmonary innate immunity. Furthermore, the presence of zebrafish CL-11 has been reported but so far fish CL-10 has not been found [65,66].

Given the important roles of these lesser known collectins in innate immunity and the fact that they are present in chicken lungs, we propose that these proteins may compensate for the loss of collagen domains among avian surfactant proteins. Hence, we hypothesized that they are present in birds in general and that they would be highly conserved. Therefore, the aims of this molecular study were: (i) to confirm lung CL-10 and CL-11 gene expression in other birds, specifically the zebra finch and turkey; (ii) to determine the presence of specific collagen functional motifs; and (iii) to undertake phylogenetic studies of these proteins in the avian lineage in the context of vertebrate collectin evolution.

## 2. Material and methods

### (a) Genome database search for PCR primer selection and gene expression analysis

Published NCBI zebra finch (*Taeniopygia guttata*) and turkey (*Meleagris gallopavo*) genome databases were queried with the amino acid sequences of chicken CL-10 (cCL-10) and CL-11 (cCL-11) using the protein BLAST tool (https://blast.ncbi.nlm.nih.gov/Blast.cgi) [67] to find orthologues in these evolutionarily distant avian species. The resultant gene sequences were used to select primers (electronic supplementary material, table S1) using primer3 software (http://bioinfo.ut.ee/primer3-0.4.0/) and NCBI primer pick (https://www.ncbi.nlm.nih.gov/tools/primer-blast/index.cgi).

Two adult birds from each species were killed by intraperitoneal injection of 60−100 mg kg^−1^ pentobarbitone sodium. Birds were humanely killed according to the animal ethics permit (U30-14), approved by the University of South Australia Animal Ethics Committee. Tissues were collected within 30 min from pulmonary and other major organs, snap-frozen and stored at −80°C for later analysis. Frozen tissues were thawed on ice to isolate RNA with an RNeasy Mini Kit 50 (Qiagen GmbH, Hilden) using a Precellys24 homogenizer (Precellys 24, Bertin Technologies, Montigny-le-Bretonneux, France). RNA quality was assessed with a NanoDrop 2000 (Thermo Scientific, Wilmington, MA). The isolated RNA (approx. 1 µg) was used to synthesize cDNA with an iScript cDNA synthesis kit (Bio-Rad Labs, Hercules, CA).

To detect and sequence the CL-10 and CL-11 genes in zebra finch (zf) and turkey (tu) lung tissue, polymerase chain reaction (PCR) was performed with cDNA and primers (electronic supplementary material, table S1) and GoTaq Green Master Mix (Promega Corp., Madison, Wisconsin, USA) according to the manufacturer’s instructions. In brief, each PCR reaction (25 µl) was performed for 3 min at 95°C, followed by 37 cycles (30 s at 95°C, 30 s annealing temperature according to electronic supplementary material, table S1, and 1 min at 72°C) and final extension for 7 min at 72°C. The PCR products were separated on an agarose (1%) gel. PCR samples with clear single bands of the expected size were sequenced (Sanger sequencing service, Australian Genomic Research Facility (AGRF), Adelaide) according to the manufacturer’s protocol using the AGRF Sanger purified PCR product sequencing method (http://www.agrf.org.au/services/sanger-sequencing). To determine the gene expression of zf and tu CL-10 and CL-11 in different tissues, a further PCR was performed on the cDNA prepared for lung, liver and kidney tissue and PCR was performed using the same conditions as described above and the product was loaded onto an agarose gel (1%) with equal amounts of sample.

### (b) Collagen functional motif search

Avian and mammalian CL-10 and CL-11 predicted protein sequences were aligned with the Clustal Omega tool (http://www.ebi.ac.uk/Tools/msa/) [68] to identify conserved collagen motifs. The conserved regions were selected based on established collagen motif size from published methods [69,70] within the size range of 9, 10 and 12 a.a. The selected motifs from aligned sequences were used as the query in the NCBI genome database with the BLASTP search tool to find cell surface receptor motifs in CL-10 and CL-11 collagen domains. To assess the reliability of selected motifs, they were compared to well-characterized human collectins SP-D and SP-A to find leukocyte-associated Ig-like receptor (LAIR) [71], osteoclast-associated receptor (OSCAR) [69] and complement motifs [42].

### (c) Protein phylogenetic analysis

Collectins and related avian lung C-type lectins [58] from the major orders of vertebrates were downloaded from the NCBI genome database (http://www.ncbi.nlm.nih.gov/genome/browse/) and aligned using the Clustal Omega tool. The aligned protein sequences were used to generate neighbour-joining phylogenetic trees (NJ trees) [72]. The phylogenetic trees generated were used to predict the evolution of these distinctive proteins in vertebrates.

## 3. Results

### (a) Confirmation of CL-10 and CL-11 expression in the avian lineage

**Figure 1.**
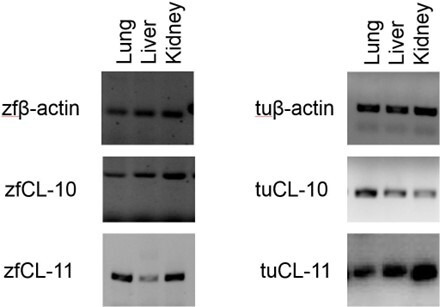
CL-10 and CL-11 gene expression in zebra finch and turkey tissues. Tissue expression of CL-10 and CL-11 mRNA (bands of approx. 400 base pairs) in lung, liver and kidney organs of the zebra finch (zf) and turkey (tu). Bands (of approx. 200 base pairs) that represent beta-actin were positive loading controls for zf and tu tissues, respectively. The expression in normal bird lungs suggests that these proteins are specialized to serve in the lung protection of both species.

An NCBI BLASTP search was performed to reveal orthologues of CL-10 and CL-11 in two phylogenetically distant avian species, the zebra finch and turkey. The BLAST search returned predicted protein and gene sequences from both species. PCR amplification and gene sequencing confirmed expression of *in silico* identified zf and tu CL-10 and CL-11 in lung, liver and kidney tissue, showing that both genes in both species were expressed in pulmonary and non-pulmonary tissues (figure 1).

**Figure 2.**
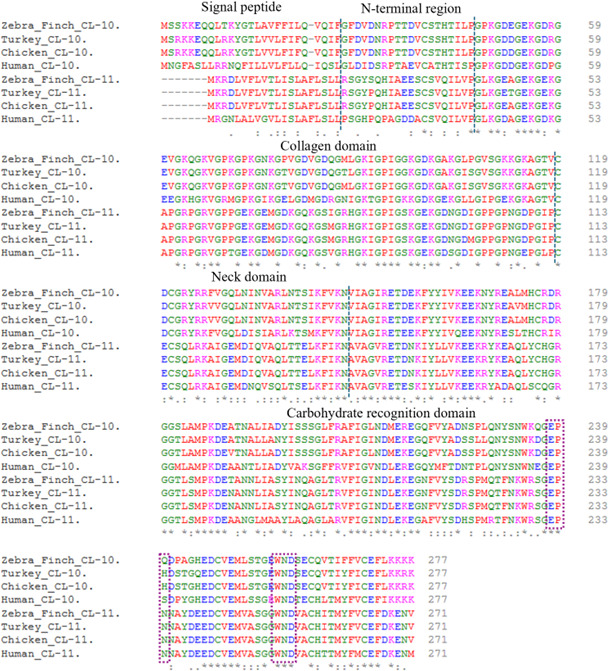
Avian and human CL-10/11 alignment showing conservation. The alignment of avian and human CL-10/11 showing conservation of a.a. sequence and similar polypeptide length suggests evolution from a common ancestor with strong purifying selection and the possibility of hybrid formation to serve similar functions from embryogenesis to various life history stages. Both proteins have retained four domains: the N-terminal, collagen, neck domain and carbohydrate recognition domain. zfCL-10 demonstrated the EPQ sequence (plum dotted box) and tuCL-10 adapted the EPH sequence compared to the EPN motif seen in CL-11 and other collectins, which would indicate a preference for mannose. However, the WND motif was conserved in all (plum dotted box). An asterisk indicates an identical amino acid, a colon indicates functional conservation reflecting amino acids with strongly similar properties and a full point (period) indicates conservation reflecting amino acids with weakly similar properties.

Sequencing of lung tissue-derived PCR products confirmed that both proteins have retained four domains (figure 2) similar to other collectins in both avian species, confirming collectin structural characteristics in avian lineages. Zebra finch CL-10 (zfCL-10) was 277 a.a. long with 92% identity to chicken CL-10 (cCL-10), 90% to turkey CL-10 (tuCL-10), 73% to human CL-10 (hCL-10) and 51% to the frog orthologue (table 1) further indicating high conservation of this protein in evolutionary distant species. Similarly, tuCL-10 with 277 a.a. showed 97% identity to the chicken and 69% to the human proteins (table 1). Not surprisingly, all structural characteristics of a C-type lectin were present in the ZfCL-10 and tuCL-10 sequences, including specific Cys residues that enable structural folding of the lectin domain and the residues involved in the orientation of the calcium ion required for ligand binding (for more details see [73]). Interestingly, the characteristic EPN motif seen in collectins, which would indicate a preference for mannose, was slightly altered because zfCL-10 demonstrated the EPQ sequence and tuCL-10 the EPH sequence (figure 2) as the respective antigen recognition motifs. Overall though, the high sequence similarity between species gives a strong indication that avian CL-10 are structurally and functionally very similar to each other and to mammalian CL-10.

**Table 1.** CL-10/11 amino acid identities among avian, human and amphibian homologues. Similarity is expressed as percentage sequence identity for the total protein/NCRD/CRD, respectively.

protein sequence	zfCL-10 (%)	zfCL-11 (%)	cCL-10 (%)	cCL-11 (%)	hCL-10 (%)	hCL-11 (%)
**cCL-10**	92/92/91				70/72/71	
**zfCL-10**		52/54/57			73/76/75	
**tuCL-10**	90/89/88		97/96/95		69/70/68	
**cCL-11**		99/99/99				
**zfCL-11**						83/86/85
**tuCL-11**		99/99/99		99/100		83/86//85
**frog CL-10**	51/76/77		67/76/76		62/71/70	
**frog CL-11**		83/83/84		83/83/84		77/78/80

Note: frog CL-10 and CL-11 sequences are based on the predicted sequences.

ZfCL-11 and tuCL-11 retained four domains comprising 271 a.a. long (figure 2) with 99% identity (267/271 a.a. identical) with cCL-11 and 83% with hCL-11 (table 1) and with identical domain sizes in zebra finch, turkey, chicken and humans confirming this protein in the avian lineage. Both proteins possessed conserved mannose-type sugar-binding EPN and WND motifs with similar neck and carbohydrate recognition domain (NCRD) lengths to the hCL-11 gene [74]. Furthermore, an NCBI BLASTP alignment of zfCL-10 and CL-11 proteins showed 52% identity (table 1) with 70% positives (i.e. amino acids with similar physicochemical properties) and no gaps.

### (b) Conservation of collagen functional motifs

The collagen domain plays an important communication role both during embryogenesis and as part of the innate immune response [41,42,75]. Hence, the conserved motifs within the collagen domains of CL-10 and CL-11 that may interact with various cell receptors were investigated. Clustal Omega aligned 29 avian CL-10 (aCL-10) and/or avian CL-11 (aCL-11) sequences both showed numerous conserved regions in the collagen domain (electronic supplementary material, figures S1 and S2). These conserved regions were selected as 9 to 12 a.a. length motifs for a BLASTP search in the NCBI genome database. The first motif from aCL-10 was ‘PGPKGDEGEK’ located at the boundary of the N-terminal and collagen domains and demonstrated high similarity to various types of collagen alpha proteins (electronic supplementary material, figure S1) (table 2). These collagen alpha proteins are structural collagens that provide mechanical strength to tissues. Furthermore, the BLASTP search using this motif as the query in the NCBI genome database showed that the root sequence ‘PGPKGD’ was conserved in fish (coelacanth), amphibians (frogs), reptiles (turtles), birds and mammals and that this site also linked the N-terminal and collagen domains of CL-10 in these vertebrates. The aCL-10 alignment showed further conserved collagen motifs: ‘GDEGEKGDRGE’, ‘GDRGEVGKQ’, ‘GKVGPKGPK’, ‘GNKGPVGDV’ and ‘GGKGDKGAKGLP’ (electronic supplementary material, figure S1), which are similar to collagen alpha type and complement proteins (table 2). Further, a specific motif identified was ‘LGKIGP’ similar to mannan-binding lectin-associated serine proteases (MASPs) ‘VGKAGP’ or ‘HGKIGP’ [77]. However, further avian-conserved motifs such as ‘GDQGMLGKIGPI’ and ‘GVSGKKGKAGTV’ when used in an NCBI BLASTP search did not return any relevant vertebrate functional protein motifs. The ‘GNKGPVGDV’ motif showed similarity to collagen structural support repeats and other proteins (table 2).

**Table 2.** Collagen domain motif similarity for CL-10 and CL-11 to various NCBI Gnomon-predicted protein sequences.

conserved collagen motif	similar to	
**CL-10 motifs**		
PGPKGDEGEK	collagen alpha-4 (IV) chain, collagen alpha-1 (VI), collagen alpha-1 (XXIII), collagen alpha-3 (V) and collagen alpha-2 (VI)	
GDEGEKGDRGE	collagen alpha−2 (XI) chain isoform 3 preproprotein [*Homo sapiens*], collagen triple helix repeat and CL-10 in many vertebrates	
GDRGEVGKQ	collagen alpha-3 of hydra, collagen alpha-1 of cotton bollworm, collagen type XI alpha-a isoform B of human	
GKVGPKGPK	identity with 8 a.a. in complement human C1q subcomponent subunit B precursor where it is located at the 20th a.a. from the start of the neck domain, human prostate collagen triple helix protein isoform b, type IV collagen alpha 4 chain, collagen alpha-2(IV) chain preproprotein and collagen alpha-1 (XXIV) chain isoform X1	
GNKGPVGDV	collagen type III alpha 1 [Salpingoeca rosetta], interferon-induced protein with tetratricopeptide repeats 1B-like [*Saccoglossus kowalevskii*], collagen-like protein, collagen alpha-1 (XXII) chain-like isoform X1 [*Oncorhynchus mykiss*] and collagen alpha-1 (XXII) chain precursor [*Danio rerio*]	
GGKGDKGAKGLP	human cell proliferation-inducing protein 41 [*Homo sapiens*], fibril-associated collagen, partial [Homo sapiens] and surfactant protein D, the SP-D ‘GDKGAKGESGLP’ motif near the neck domain.	
GEKGDRGEV	human complement C1q tumour necrosis factor-related protein 1, macrophage receptor, collagen type VII, XVIII and putative GPCR interacting protein GIP	
**conserved collagen motif**	**similar to**	
**CL-11 motifs**		
GLKGEAGEKGEK	to the motifs of human collagen alpha-1 type XV, type XXIII alpha 1 isoform CRA_b, collagen alpha−2 (VI) chain isoform 2C2a precursor and otolin-1 precursor	
GAPGRPGRVGPP	human collectin-11 isoform f precursor, C1q-related factor precursor and complement C1q-like protein 4 precursor	
GPPGEKGEM	C1q-related factor precursor from the C1q/TNF family [76], complement C1q-like protein 3 precursors, endothelial cell apoptosis protein E-CE1, collagen alpha-1 (V), macrophage scavenger receptor types I and II isoform type 1, collagen alpha-1 (IV) chain isoform 1 preproprotein; and collagen alpha-1 (XVII) chain	
GDKGQKGSI/M	Similar to hCL-11 ‘GDKGQKGSV’ motif	
GKIGPIGSKGEK	ficolin, collectin sub-family member 11, isoform CRA_a, macrophage receptor, collectin-10 isoform 1 precursor and collectin 34	
GPPGPNGDPGIP	similar to hCL-11 ‘GPPGPNGEPGLP’ motif	

Similar BLASTP search criteria were applied to find conserved aCL-11 collagen domain motifs with known amino acid sequences and Clustal Omega similarity. The search results revealed the following motifs: ‘GLKGEAGEKGEK’, ‘GAPGRPGRVGPP’, ‘GPPGEKGEM’, ‘GDKGQKGSI/M’, ‘GKIGPIGSKGEK’ and ‘GPPGPNGDPGIP’ (electronic supplementary material, figure S2). These short amino acid motifs were used in an NCBI BLAST search and resulted in similar motifs in various proteins in various species, as presented in table 2. Additional motif searches based on the known PGP triplet that supports the OSCAR receptor [69] in aCL-11 revealed the presence of ‘GPPGPNGDP’ and/or ‘GPPGPNGDPGIP’ (electronic supplementary material, figure S2). An alignment of aCL-11 (approx. 41 species) with hSP-D and hCL-11 revealed the ‘GPPGPNGDP’ motif (electronic supplementary material, figure S2), which was similar to the hSP-D OSCAR ‘GPPGPPGVP’ motif [69]. Furthermore, conserved motif searches were conducted in CL-10/11 multiple sequence alignments to find the known complement GEK triplet sequence [42]. The GEK search identified ‘GEKGDRGEV’ that acts as an alternative start to the ‘GDEGEKGDRGE’ motif. Another motif, ‘HGKIGP’, which was identical to MASP ‘HGKIGP’ or similar to ‘VGKAGP’ was also identified [77]. Further proteins with similar motifs are presented in table 2.

### (c) Protein phylogenetic analysis

**Figure 3.**
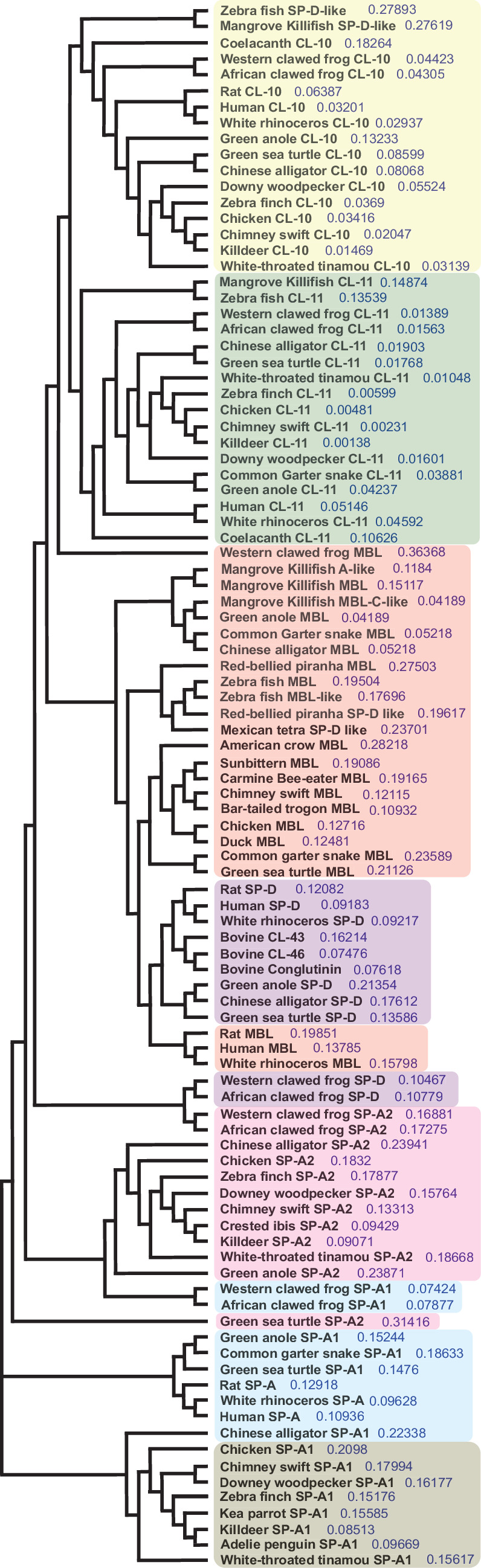
Neighbour-joining tree of vertebrate collectins. CL-10 and -11 form separate clades that were connected to MBL and other collagenous and non-collagenous lectins. The nodes of each protein were highlighted and appear in order: CL-10 (yellow), CL-11 (green), MBL (red), SP-D (purple), SP-A2 (pink), non-avian SP-A1 (blue) and aSP-A1 (grey)—noting that the MBL and SP-A2 nodes are not entirely contiguous. The values shown in the tree represent the ‘length’ of the branches, which is an indication of the evolutionary distance between the sequences (https://ebi-biows.gitdocs.ebi.ac.uk/documentation/faqs/clustal/).

A phylogenetic tree based on CL-10, CL-11 and related lung-associated C-type lectin protein sequences from air-breathing vertebrates was developed using an NJ tree as implemented in the Clustal Omega software package [72]. The resultant tree (figure 3) revealed separate clades of orthologous branches for each of the genes and consisted of most major vertebrate lineages. Both the CL-10 and CL-11 genes formed separate clades from a common node, each including the major vertebrate groups of fish, amphibians, reptiles, birds and mammals. Most MBL and SP-D genes formed separate clades of orthologous vertebrate branches and were joined by a common MBL/SP-D node. In addition, the SP-D clade included the conglutinin and CL-43 genes. The exception was NCBI GNOMON predicted frog SP-D, which formed a separate clade with frog SP-A2 that together were most closely related to the MBL/SP-D/conglutinin/CL-43 clade. The CL-10/11 and MBL/SP-D/conglutinin/CL−43 nodes together were connected first to the frog SP-A2/SP-D node, followed by the crocodilian, reptilian and avian SP-A2 and the amphibian, reptilian, mammalian, crocodilian and avian SP-A1 nodes. Finally, all these nodes were linked to the clade consisting of alligators and avian SP-A1, which served as an out-group.

## 4. Discussion

A bioinformatics search revealed the predicted CL-10 and CL-11 protein sequences with high homology in more than 40 avian species (electronic supplementary material, figures S1 and S2), which suggested that they are highly conserved in the avian lineage. PCR sequencing confirmed zf and tu CL-10/11 gene expression in normal lungs, with high sequence identity to chicken (approx. 90%) and humans (approx. 70%) (table 1). The high sequence identity was also reflected in collagen motif conservation, which supports a function for these proteins in birds involving innate immune interactions. Furthermore, the sequence homology as well as the presence of predicted gene sequences in fish (CL-11 only), frogs, reptiles and mammals suggest evolution from a common ancestor. Phylogenetic analysis suggested that MBL was the likely ancestor of CL-10/11 (figure 3).

### (a) Conservation and innate immune functions of CL-10/11

Chicken CL-10 and CL-11 gene expression has been demonstrated in adult chicken lungs [36], and it was moderately increased in response to IBV and *E. coli* [64] and avian influenza virus (AIV) infection [78]. The moderate increase in response to bacterial and viral infection supports a role for these proteins in pulmonary innate defence in the chicken. Similar to the adult chicken, both zebra finch and turkey normal adult lung tissue demonstrated CL-10 and CL-11 gene expression (figure 1). Immunohistochemistry showed a differential localization of CL-10 and CL-11 in human tissues [79]. While alveolar macrophages contain both collectins, CL-11 was also found in type 1 and type II epithelial cells. Since type II cells are strong secretory cells known for their production and secretion of pulmonary surfactant, it is possible that CL-11 homotrimers are present in the alveolar lining fluid of mammalian lungs. Mammalian CL-10 and CL-11 were shown to exist in plasma as disulfide bridge-stabilized heterotrimers consisting of one CL-10 and two CL-11 monomers [60]. In chickens, surfactant protein A was localized in the alveolar lining fluid and to specific epithelial cells in the atria, likely resembling the mammalian type II cells [63]. It would be interesting to determine if these cells in the avian lung also produce avian CL-10 and/or CL-11 and if they are assembled as hetero- as well as homotrimers. Considering the CL-10 and CL-11 gene expression in the lungs, together with their high amino acid sequence identity (figure 2), it is likely that these proteins serve a common function in avian lung protection. In addition to chicken respiratory pathogen infection studies, hCL-10 function has been characterized with respect to sugar binding, but antigen-binding studies have not been performed [62]. The hCL-10 sugar-binding analyses demonstrated a weaker binding to D-mannose, N-acetylglucosamine, D-galactose and D-fucose [33] compared with CL-11, but the *E. coli* binding studies were inconclusive [80]. Hence, the response of CL-10 to human lung infection is unknown. However, further investigations showed that CL-10 has important roles in blood circulation, complement activation [60], embryonic development and regulation of craniofacial development, with its deficiency leading to the developmental abnormalities seen in human 3MC syndrome [61,81]. Mutations in CL-11 are also linked to 3MC syndrome [59]. The high sequence identity (table 1) and similar peptide length (figure 2) between humans and aCL-10 suggest that they can form similar structures and perform similar functions. However, aCL-10 adopted an EPH/Q a.a. sequence (i.e. glutamic acid, proline and either histidine or glutamine) as its ligand or carbohydrate-binding motif, compared with an EPS (i.e. glutamic acid, proline, serine) sequence in hCL-10. This adaptation appears to be unique to avian species as a search for similar motifs in major orders of vertebrates returned no collagenous lectins. The EPH/Q adaptation ensures diversity and also eliminates duplication in antigen motifs (e.g. with respect to EPS of avian SP-A2 (aSP-A2)) [58] at the pulmonary surface. However, the precise function of aCL-10 with an EPH/Q motif was unknown; we postulate that this adaptation may serve avian embryonic development and innate immune protection from a wide range of air-associated pathogens, which needs to be investigated.

Human CL-11 with its EPN motif has been well characterized with more structural and functional studies compared with hCL-10 [80,82]. The crystallographic structure of the CL-11 NCRD trimer [82] revealed strong similarity to other collectins such as SP-D [83] and MBL [84]. This similarity strengthens the suggestion for a role of the EPN motif in mannose-type sugar binding that defines antigen pattern recognition [73,85]. The hCL-11 sugar-binding studies showed its preference for D-mannose, N-acetylmannosamine and fucose [33], as demonstrated by binding to influenza A virus (IAV), *E. coli*, *P. aeruginosa* [34,74] and apoptotic cells [60,74,86]. Recent investigation showed that CL-11 promotes invasion and injury of respiratory epithelial cells by SARS-CoV-2 indicating an unknown function in COVID-19 [87]. These studies support a broad function for CL-11 in human innate immunity similar to that of MBL [88,89] and SP-D [46]. It is likely that comparable functions may be expected of aCL-11 due to the conservation of key antigen recognition motifs EPN and WND in the CRD as well as specific motifs in the collagen domain.

Interestingly, hCL-10 forms hybrids with hCL-11 [60], which may alter antigen binding and myeloid cell interaction due to increased sequence diversity of the NCRD and collagen domain motifs. The similar size and conservation of cCL10 and cCL-11, coupled with a similar localization [36], suggest that CL10/CL11 hybrid formation may also occur in chickens; however, this remains to be investigated. Importantly, chickens do possess a leukocyte receptor cluster that is involved in the immune response [90].

### (b) The role of the collagen domain

Besides the simple structural function of the collagen domain to stabilize trimers and position the lectin domains, several other functions have been assigned to specific collagen motifs in mammalian collectins. These include sequences required for complement activation via MASPs [91], and interaction with myeloid cells [17,48,92], via receptors such as LAIR [71], and OSCAR. The bioinformatics search conducted in this study revealed that avian CL-10 and CL-11 contain many of these conserved collagen domains related to inflammation and complement activation (electronic supplementary material, figures S1 and S2). Although it is very suggestive that these motifs have similar functionality as their mammalian counterparts, specific functional studies including mutagenesis should be performed to determine their true role in innate immunity.

Human CL-11 plays major roles in embryogenesis [59], uptake of apoptotic cells by phagocytic cells, cell debris clearance by complement [60] and inflammation regulation [33,74]. Immune functions such as myeloid cell interaction and complement activation require conserved collagenous domain motifs and the hCL-11 collagen motifs are unknown. CL-11 is recruited to bind ligands by C1q/TNF-related protein 6 which is a pattern recognition molecule to activate the complement system [93]. Furthermore, collectin protein innate immune functions showed the collagen domain interaction with myeloid cells [17,48,92], complement activation via mMASPs [91] and some collagen motifs such as the complement motif.

A bioinformatics search revealed high sequence similarity of these collectins to chicken and mammalian species, indicating significant evolutionary conservation (electronic supplementary material, figures S1 and S2). Earlier investigations of CL-10/11 reported conservation between vertebrate species, the importance of their ancestral role and suggested evolution by mutual gene duplication from a common ancestor [62] and exon shuffling [80]. Specifically, the expression of CL-11 collagen domain variants by exclusion or inclusion of exons (e.g. short collagen domain of 42 a.a. versus 66 a.a. in full length) [62] indicates the evolution of collectin family members by exon addition or deletion or exon shuffling by recombination.

### (c) Evolution of CL-10/11 among air-breathing vertebrates

To resolve the evolution of these proteins among air-breathing vertebrates, a phylogenetic tree was constructed. The formation of separate clades consisting of orthologous branches of vertebrate CL-10 and CL-11 genes (figure 3) suggests a gene duplication event at the common CL-10/CL-11 node and speciation to yield two distinct genes with different but complementary protein functions (e.g. different antigen-binding sites), followed by significant purifying selection throughout the radiation of the vertebrates. The closest neighbour to the CL-10/CL-11 node was the MBL node, including fish (mangrove killifish and zebrafish) and frog MBL. This suggests that one of either CL-10 or CL-11 arose by gene duplication from an early MBL ancestor and that this was followed by a second gene duplication event to yield both CL-10 and CL-11. In turn, the fish and frog MBL node was rooted to the node of the remaining vertebrate (i.e. avian, reptilian and mammalian) MBL genes, which clustered with the vertebrate SP-D and mammalian conglutinin and CL-43 genes. The main MBL/SP-D/conglutinin/CL-43 node, in turn, was directly rooted to frog SP-A2 and SP-D and this node, in turn, was rooted to crocodile, reptilian and avian SP-A2 followed by frog, reptilian, avian and mammalian SP-A1, which represent the most derived genes in this family. The fact that frog SP-A2 and SP-D form a separate clade from all other collectins suggests that these proteins were the ancestors for all the described collectins in this tree, yet their common ancestor that predates the tetrapods is still unknown [58]. Finally, the evolution of both non-collagenous and short collagenous avian lectins as an out-group that was linked by reptilian and mammalian collagenous SPs to the large collagenous lectins (e.g. SP-D) indicates that collectins evolved most likely from ancestral proteins such as non-collagenous C-type lectins [94] or invertebrate C-type lectins [95] lacking the collagen domain.

## 5. Conclusion

Considerable CL-10 and CL-11 gene expression was detected in the lungs of adult turkeys and zebra finches, and bioinformatics revealed that CL-10 and CL-11 were highly conserved among birds and other vertebrates. In mammalian lungs, collagen-like domains in SP-D and SP-A variants harbour binding sites for immune-related receptors such as MASP and OSCAR. The conservation of collagen-like domain motifs in aCL-10/11, containing similar putative binding sites for immune-related receptors, may compensate to some extent for the absence of SP-D, the partial loss of a collagen-like domain in aSP-A1 and the absence of a collagen-like domain in SP-A2 in the small and rigid avian parabronchial lungs. However, immunohistochemistry localization studies in avian lungs will be necessary for a better understanding of their roles in the alveolar microenvironment.

## Data Availability

Sequence data are provided in supplementary figures [96].
